# High versus standard blood pressure target in hypertensive high-risk patients undergoing elective major abdominal surgery: a study protocol for the HISTAP randomized clinical trial

**DOI:** 10.1186/s44158-023-00133-3

**Published:** 2023-12-01

**Authors:** Antonio Messina, Andrea Cortegiani, Stefano Romagnoli, Giovanni Sotgiu, Federico Piccioni, Katia Donadello, Massimo Girardis, Alberto Noto, Salvatore Maurizio Maggiore, Massimo Antonelli, Maurizio Cecconi

**Affiliations:** 1 Department of Biomedical Sciences, Humanitas University, Via Rita Levi Moltancini 4, Pieve Emanuele (MI), 20072 Italy; 2 IRCCS Humanitas Research Hospital, Department of Anesthesia and Intensive Care Medicine, Via Alessandro Manzoni, 56, Rozzano (MI), 20089 Italy; 3 Department of Surgical Oncological and Oral Science, University of Palermo, Department of Anesthesia Intensive Care and Emergency, Policlinico Paolo Giaccone, Palermo, Italy; 4 Department of Anesthesia and Intensive Care, University of Florence, Azienda Ospedaliero-Universitaria Careggi, Florence, Italy; 5 Clinical Epidemiology and Medical Statistics Unit, Department of Biomedical Sciences, University of Sassari, Sassari, Italy; 6 Department of Surgery, Dentistry, Gynecology and Paediatrics, University of Verona, Verona, Italy; 7 Department of Anesthesia and Intensive Care, University Hospital of Modena, Modena, Italy; 8 Department of Human Pathology of the Adult and Evolutive Age “Gaetano Barresi”, Division of Anesthesia and Intensive Care, University of Messina, Policlinico “G. Martino”, Messina, Italy; 9 University of Chieti-Pescara and Clinical, Department of Anesthesia, Critical Care and Pain Medicine, SS. Annunziata Hospital, Chieti, Italy; 10 Department of Emergency, Anesthesiology and Resuscitation Sciences, Fondazione Policlinico Universitario Agostino Gemelli IRCCS, 00168 Rome, Italy

**Keywords:** Intraoperative hypotension, Blood pressure, Fluid therapy, Hemodynamic monitoring, Postoperative complications, Organ injury

## Abstract

**Background:**

The intraoperative period is often characterized by hemodynamic instability, and intraoperative hypotension is a common complication. The optimal mean arterial pressure (MAP) target in hypertensive patients is still not clear. We hereby describe the protocol and detailed statistical analysis plan for the high versus standard blood pressure target in hypertensive high-risk patients undergoing elective major abdominal surgery: the HISTAP randomized clinical trial. The HISTAP trial aims at addressing whether the use of a higher intraoperative MAP target in high-risk hypertensive surgical patients scheduled for elective abdominal surgery would improve postoperative outcomes, as compared to the standard and recommended perioperative MAP, by using a composite outcome including a 30-day mortality from surgical intervention and at least one major organ dysfunction or new onset of sepsis and septic shock occurring 7 days after surgery.

**Methods:**

The HISTAP trial is an investigator-initiated, pragmatic, parallel-grouped, randomized, stratified, analyst-blinded trial with adequate allocation sequence generation, and allocation concealment. We will allocate 636 patients to a MAP target ≥ 80 mmHg (treatment group) or to a MAP target ≥65 mmHg (control group). The primary outcome is a composite outcome including a 30-day mortality from the operation and major organ complications. Secondary outcomes are mortality at 30 days, intensive care unit (ICU) length of stay, ICU readmission, Sequential Organ Failure Assessment (SOFA) scores recorded up to postoperative day 7, overall intraoperative fluid balance, vasopressors use, and the need for reoperation. An unadjusted *χ*^2^ test will be used for the primary outcome analysis. A Cox proportional hazards model will be used to adjust the association between the primary outcome and baseline covariates.

**Conclusions:**

The HISTAP trial results will provide important evidence to guide clinicians’ choice regarding the intraoperative MAP target in high-risk hypertensive patients scheduled for elective abdominal surgery.

**Supplementary Information:**

The online version contains supplementary material available at 10.1186/s44158-023-00133-3.

## Introduction

The intraoperative period is often characterized by hemodynamic instability and intraoperative hypotension is a common complication. Various definitions of intraoperative hypotension have been evaluated in the literature, resulting in a widely varying incidence of hypotension (5 to 75%) [1, 2].

Hypotension is associated with worse clinical outcomes, and this is supported by robust evidence. For instance, Walsh et al. obtained perioperative data for 33,330 non-cardiac patients and found that even short periods of intraoperative hypotension (MAP below 55 mmHg) were associated with acute kidney injury (AKI) and myocardial injury [3]. In another study, Sun et al. conducted a retrospective cohort study of 5127 patients undergoing noncardiac surgery and found that postoperative AKI was associated with sustained intraoperative periods of MAP less than 55 and less than 60 mmHg [4]. More recently, the same authors found that the incidence of stroke after cardiac surgery requiring cardiopulmonary bypass was strongly associated with sustained MAP < 64 mmHg [5]. Finally, a systematic review of 42 studies summarized reported risks of myocardial injury, acute kidney injury, and death depending on the severity and duration of intraoperative hypotension [6]. The risk of any end-stage organ injury was slightly increased when MAP was sustained at less than 70 mmHg for just 10 min. The risk was moderately increased with exposures to MAP less than 65 to 60 mmHg for at least 5 min, or any exposure to MAP less than 55 to 50 mmHg. A high risk of any end-stage organ injury was reported for exposures to MAP less than 65 mmHg for at least 20 min, MAP less than 50 mmHg for at least 5 min, or any exposure to MAP less than 40 mmHg [6].

As a matter of fact, keeping MAP > 65 mmHg during the intraoperative period is considered a key target to reduce postoperative complications and this threshold is nowadays considered a standard of good clinical practice in the intraoperative period [7, 8].

To date, there is no clear evidence regarding the clinical benefit of maintaining a higher MAP target in hypertensive patients. In fact, in patients with chronic arterial hypertension, blood flow autoregulation curves are shifted to the right, toward higher blood pressures [8–10]. Therefore, patients with chronic arterial hypertension possibly less tolerate hypotension than normotensive patients and may need higher perioperative blood pressures [8, 10].

This assumption is not supported by robust evidence obtained from large randomized controlled trials (RCT), insofar. For instance, a single-center RCT on 458 patients scheduled for non-cardiac surgery, showed no effect of the intraoperative MAP target of ≥60 mmHg or ≥ 75 mm Hg on clinical outcomes; however, most of the patients were not hypertensive at home [11]. Finally, in a large and recent RCT in a 2×2 factorial design assessing the effect of tranexamic acid on postoperative bleeding, patients were given chronic antihypertensive medications and MAP ≥ 60 mmHg was targeted intraoperatively vs. a hypotension-avoidance strategy where antihypertensives were only given if hypertensive prior to surgery and MAP ≥ 80 mmHg was targeted intraoperatively. This strategy did not affect 30-day major vascular complications [12].

### Trial aim

The HISTAP trial will evaluate if a higher intraoperative MAP target (≥80 mmHg—treatment group) in high-risk hypertensive surgical patients scheduled for elective abdominal surgery would improve postoperative outcome (evaluated by using a composite outcome including a 30-day mortality from surgical intervention and at least one major organ dysfunction or new onset of sepsis and septic shock occurring 7 days after surgery), in comparison with the recommended perioperative MAP range (≥65 mmHg—control group).

## Methods

### Design

The HISTAP trial is a pragmatic, parallel-grouped, randomized, stratified, analyst-blinded trial with allocation sequence generation and concealment. The HISTAP trial is co-sponsored by Humanitas Research Hospital; Rozzano – Milano and the Società Italiana di Anestesia Analgesia Rianimazione e Terapia Intensiva (SIAARTI). The Steering Committee will grant authorship depending on personal involvement according to the Vancouver definitions. A group authorship (“SIAARTI Study Group”) will be created, including all the investigators of the participating centers, according to predefined rules for authorship (see Supplementary materials).

### Trial interventions and groups

After the randomization, patients will be assigned to two groups according to different MAP targets during the intraoperative period:

- Intervention group: target of intraoperative MAP ≥80 mmHg
- Control group: target of intraoperative MAP≥65 mmHg

### Trial conduct

The protocol has been prepared according to the Guidelines for Inclusion of Patient-Reported Outcomes in Clinical Trial Protocols: The SPIRIT [13], to the Helsinki principles of good clinical practice in its latest version [14], to the international guidelines for good clinical practice (GCP) [15], and to Italian laws. The Ethical Committee of the coordinator center (Humanitas Research Hospital; Rozzano - Milano) approved the protocol study (Autorization n 3392/15 November 2022; Protocol Number 937/22), which will be approved by local ethical committees of participating centers.

The trial has been prospectively registered at ClinicalTrials.gov as NCT05637606.

### Randomization

Eligible patients will be assigned in a 1:1 ratio to either control or treatment group. A randomization list will be created by a computer with the use of a permuted block design and embedded in the eCRF. Randomization will be performed using a block of 6 and stratified according to predefined baseline characteristics:

1. Age ≥ 75 years (stratification variable)
2. Preoperative systolic arterial pressure (SAP) at the timing of preoperative visit (stratification variable)
  - < 140 mmHg
  - ≥ 140 mmHg

On the day of surgery, the results of the randomization will be returned by the eCRF once the inclusion and exclusion criteria have been confirmed and the anesthesiologist in charge in the operating room will follow the assigned group. Data will be collected intraoperatively and during all the postoperative period in the eCRF. Enrollment, randomization, and data collection will be managed using REDCap [16, 17] electronic data capture tools hosted at the Italian Society of Anaesthesiology, Analgesia, Resuscitation and Intensive Care (SIAARTI) (see Supplementary Materials for further details).

### Blinding

Trial intervention is not blinded for investigators in the operating room and patients, being blinding to intraoperative MAP target is unfeasible. The statistician will be blinded to allocation. Clinical outcomes will be recorded by personnel blinded to patients’ allocation.

### Inclusion criteria

(all the following)

1. Adult patients ≥ 60 years.
2. History of chronic hypertension requiring home therapy.
3. Scheduled for major elective abdominal surgery (laparoscopic, robotic, or laparotomic).
4. Expected surgical duration of at least 3 h (planned surgical time).
5. Needing invasive arterial and hemodynamic monitoring as decided by the attending anesthetist, according to the rules of good clinical practice of each involved center.

AND

At increased risk of postoperative complications

(at least one of the following):

1. American Society of Anesthesiologists (ASA) classes 3 or 4.
2. Known or documented history of coronary artery disease (angina, myocardial infarction, or acute coronary syndrome).
3. Known or documented history of peripheral vascular disease.
4. Known or documented history of heart failure requiring treatment.
5. Ejection fraction less than 30% (as defined by preoperative echocardiography).
6. Signs of diastolic moderate to severe dysfunction or chronic hypertensive cardiomyopathy (as defined by preoperative echocardiography).
7. Moderate or severe valvular heart disease (as defined by preoperative echocardiography).
8. Diagnosis of Chronic Obstructive Pulmonary Disease (COPD) Radiographically confirmed or according to Global Initiative for Obstructive Lung Disease (GOLD) criteria.
9. Diabetes currently treated with an oral hypoglycemic agent and/or insulin.
10. Morbid obesity (body mass index ≥ 35 kg/m^2^).
11. Preoperative serum albumin <30 g/l.
12. Anaerobic threshold (if done) <14 ml/kg/min.
13. Exercise tolerance is equivalent to six metabolic equivalents (METs) or less.

### Exclusion criteria

1. Refusal of consent.
2. Chronic kidney disease with glomerular filtration rate <30 ml/min/1.73 m^2^ or requiring renal-replacement therapy for end-stage renal disease.
3. Acute cardiovascular event, including acute or decompensated heart failure and acute coronary syndrome (within the prior 30 days).
4. Urgent or time-critical surgery.
5. Aortic or renal vascular surgery*.
6. Liver surgery**.
7. Neurosurgery.
8. Surgery for palliative treatment only or ASA class 5.
9. Pregnancy.

*Monolateral partial renal resections can be included; nefrectomies are excluded

**Focal wedge metastastasecotomies can be included

### Fluid management

In both groups, we required the use of balanced crystalloid solution as the routine IV fluid therapy in this study. In each group, patients will receive 5 ml/kg/h for laparotomic surgery and 3 ml/kg/h for laparoscopic surgery as standard maintenance fluid infusion during surgery. These rates can be modified by the attending anesthetists if he/she judges them inadequate to maintain the desired organ perfusion and fluid balance, or because of hemodynamic signs of suspected hypovolemia, as suggested by the hemodynamic monitoring.

Fluids can also be used infused as a fluid challenge (FC) of 4 ml/kg of balanced crystalloid solutions within 10 min, to revert an episode of hypotension, managed according to the algorithms reported in the Supplementary Materials for laparotomic and laparoscopic patients.

These algorithms consider baseline pre-FC values of pulse pressure variation (PPV) or stroke volume variation (SVV) and/or the response to a mini fluid challenge (Mini-FC) test [18] for guiding fluid boluses administration.

A patient is considered responder to the FC for a SV or SVI increase of at least 10% from baseline, after FC infusion [19]. A mini-FC is considered positive for a SV or SVI increase of at least 5% from baseline [18]. The same protocol used during laparoscopy will be adopted in conditions associated to PPV and SVV unreliability, such as in patients with atrial fibrillation or for those patients with recurrent intraoperative extrasystoles (See Supplemental Materials for further details).

### Pressure management

In both groups, an episode of hypotension is managed according to the algorithms reported in the Supplementary Materials for laparotomic and laparoscopic patients, respectively.

In both groups, the target MAP can be maintained by means of boluses of vasoactive agents ephedrine (2.5 mg) or etilefrine (1 mg) or a continuous infusion of norepinephrine, as decided by the attending anesthetist.

The continuous infusion of norepinephrine may start at the induction of general anesthesia or during the intraoperative period, according to the clinical condition of the patient and the predicted risk of hemodynamic instability. The maximal dose for the ephedrine allowed is 25 mg (10 boluses of 2.5 mg). The maximal dose for the etilefrine allowed is 10 mg (10 boluses of 1 mg). Above these doses, a continuous infusion of norepinephrine is started in both groups. The starting dose of norepinephrine is the lowest needed to reach the predefined MAP target, with an increase of 0.05 mcg/kg/min, as decided by the attending anesthetist to keep the MAP within the predefined ranges.

### Intraoperative management: general policy

1. General anesthesia induction and maintenance will be performed according to the standard clinical practice of each center. Induction will be performed with the use of propofol and/or benzodiazepines, remifentanil or other opiates, and neuromuscular blockade, as considered appropriate to the attending anesthesiologist. Inhaled or intravenous anesthetics will be used for maintenance of general anesthesia, at the discretion of the attending anesthesiologist, according to hemodynamic parameters or to target neurological monitoring within normal ranges, if available.
2. Mechanical ventilation is delivered with the use of a tidal volume between 6 and 8 ml/kg of predicted body weight, with a suggested positive end-expiratory pressure (PEEP) between 5 and 10 cmH_2_O, an inspired oxygen fraction (FIO_2_) to maintain oxygen saturation **≥** 95% and the respiratory rate adjusted to maintain end-tidal carbon dioxide concentration between 30 and 35 mmHg. The PEEP choice is at the discretion of the attending physician, considering laparotomic/laparoscopic surgery, the position of the patient, and the mechanical proprieties of the respiratory system.
3. Hemodynamic monitoring: The patients will be equipped with hemodynamic monitoring based on invasive arterial waveform signal analysis used as a routinely standard of care and already available in the center (no adjunctive costs).
4. After the induction, invasive blood pressure measurement will be started as soon as possible. Non-invasive blood pressure measurement will be set at 3 min until the invasive measurement is available.
5. Core temperature maintenance according to the standard clinical practice of each center.
6. Preoperative or intraoperative use of epidural or spinal analgesia for postoperative pain control is allowed.
7. All patients are managed with the same red cell transfusion trigger of 7.0 g/L, but this could be modified after assessment of cardiovascular risk or concern for active bleeding.

Further specific and operative considerations on intraoperative fluid and pressure management are reported in the Supplementary Materials.

### Outcome measurement

#### Primary outcome

Composite outcomes include a 30-day mortality from surgical intervention and at least one major organ dysfunction (renal, respiratory, cardiovascular, and neurologic system) or new onset of sepsis and septic shock occurring 7 days after surgery (definitions of major complications in the Supplementary Materials).

The occurrence and severity of organ dysfunction will be assessed at least once daily and during the follow-up. Patients will be followed up for 30 days after the surgical intervention. Patients will be contacted at 30 days either by telephone for those who are discharged or during a medical visit for those who are not discharged. Follow-up-related variables are described in the Supplementary Materials.

#### Secondary clinical outcomes

- Hospital stay (days)
- Mortality at 30 days
- Intensive care unit (ICU) stay (days)
- ICU readmission
- Sequential Organ Failure Assessment (SOFA) scores recorded up to postoperative day 7
- Overall intraoperative fluid balance, including intraoperative infusions (crystalloids, colloids, blood products) and intraoperative loss balance (urine output and blood loss)
- Vasopressor use
- Need for reoperation

### Statistical analysis

#### Sample size

The sample size has been calculated considering the occurrence of death rate and major organ adverse events in previous randomized controlled trials (RCT). We selected from previous metanalyses [20, 21] those studies published in the last 10 years, enrolling more than 100 high-risk patients (i.e., with previous cardiovascular diseases or hypertensive > 50% or ASA II > 50%) with a mean age > 65 years (Table 1). Finally, we added the recent RCT on the effect of individualizing blood pressure targets [22] (Table 1). Considering the incidence of major events in the literature for the selected population, we calculated that a sample of 636 patients would provide the trial with 90% power to detect an absolute difference of 12.5% with respect to the primary outcome, at a 2-sided *α* level of .05, assuming an event rate of 41% in the composite outcome in the standard treatment group. This number is considered 5% of dropouts.

**Table 1:** Studies evaluated for sample size calculation

Authors	Patients (*n*)		Mortality (*n*)		Events (*n*) (overall)		Major events (*n*) (overall)	
	Case	Control	Case	Control	Case	Control	Case	Control
Brandstrup et al. [23]	72	69	0	4	21	40	8	18
Gao et al. [24]	86	93	2	4	46	84	18	23
Abraham-Nordling et al. [25]	82	79	NA	NA	50	81	22	39
Kalyan et al. [26]	121	118	2	4	102	115	46	46
Jie et al. [27]	96	89	0	0	60	97	19	21
Grant et al. [28]	164	166	1	1	96	100	35	46
Myles et al. [29]	1493	1490	96	95	823	676	549	437
Wuethrich et al.	83	83	0	0	77	161	16	29
Benes et al. [30]	60	60	1	2	32	73	13	41
Pearse et al. [31]	62	60	6	7	43	90	28	47
Pearse et al. [32]	368	366	12	11	134	158	342	349
Futier et al. [22]	149	146	9	8	169	210	56	75

*N* number of patients

The choice of 12.5% as an expected difference in the primary outcome is based on the effect size observed in a previous randomized-controlled study [32].

#### Statistical analysis

Demographic and baseline disease characteristics will be summarized with the use of descriptive statistics. According to types of variables and data distribution, sample characteristics will be summarized using measures of central tendency (mean or median), variability (standard deviation/SD or interquartile ranges/IQR), and frequency distributions. Shapiro-Wilk test will assess the normality of data distribution. All analyses will be conducted before the randomization code is broken, in line with the International Conference on Harmonization Good Clinical Practice guidelines. The primary and secondary outcomes will be analyzed under several different analysis set definitions as described below (i.e., primary and secondary outcomes section).

The analyses set for testing study outcomes are in the intention-to-treat (ITT) approach, defined as all randomized participants for whom there is consent for the use of data. The conclusion of the trial will be based on the ITT analysis. The per-protocol population is defined as the ITT population except those having one or more major protocol violations. Concerning missing data, in general for primary and secondary outcomes, they will be not imputed. Missing data for study outcomes will be reported in the results report. Furthermore, once the data is collected and its quality is evaluated, a potential application of the Markov Chain Monte Carlo method will be evaluated for missing data imputation.

#### Primary outcome analysis

An unadjusted *χ*^2^ test will be used for the primary outcome analysis. A Cox proportional hazards model will be used to identify relevant baseline covariates associated with the primary outcome.

Results for the primary outcome will be additionally reported as absolute and relative risks with 95% confidence intervals. Kaplan-Meier curves will be plotted for the overall primary outcome and all its components and compared by the marginal Cox model. Follow-up time will be censored at 30 days following surgery. The time to death or organ dysfunction (whatever comes first) will be analyzed using a marginal Cox proportional hazards model with results reported as hazard ratios with 95% confidence intervals, and the proportional hazard assumption will be verified using the Schoenfeld test and plotting residuals. All hypothesis tests will be 2-sided, and *p* <0.05 will be considered statistically significant.

#### Secondary outcome analyses

The secondary outcomes analysis will be performed in conjunction with the primary outcomes analysis. Statistical methods for testing multiple outcomes will include descriptive and inferential techniques (parametric or non-parametric approach will be conducted according to normal distribution assessment). All hypothesis tests will be 2-sided, and *P* <0.05 will be considered statistically significant.

- Hospital stay (days): it will be described using mean (SD) or median (IQR), this measure will be compared between groups using unpaired Student’s *t*- or Mann-Whitney *U* tests.
- Mortality at 30 days: it will be described using absolute and relative (percentages) frequencies, it will be compared between groups of interest (i.e., sex, age classes) using Pearson chi-square or Fisher’s exact tests.
- Intensive care unit (ICU) stay (days): it will be described using mean (SD) or median (IQR), this measure will be compared between groups using unpaired Student’s *t* or Mann-Whitney *U* tests.
- ICU readmission: proportion of patients re-admitted to ICU will be described with absolute and relative (percentages) frequencies, it will be compared between groups of interest using Pearson chi-square or Fisher’s exact tests.
- Sequential Organ Failure Assessment (SOFA) scores recorded up to postoperative day 7: SOFA scores will be summarized using median and IQR, their differences between groups will be evaluated using the Mann-Whitney *U* test (for unpaired data) or by Wilcoxon test (for matched data: repeated measures for repeated time-points).
- Overall intraoperative fluid balance, including intraoperative infusions (crystalloids, colloids, blood products) and intraoperative loss balance (urine output and blood loss).
- Dose and timing of vasoactive drug infusion intraoperatively (dose will be described using mean (SD) or median (IQR), they will be compared between groups using unpaired Student’s *t* or Mann-Whitney *U* tests). In case of evaluation of these parameters in different timepoints (i.e., repeated measures/comparison from baseline to follow-up), comparisons of their differences will be evaluated using paired Student’s *t* or Wilcoxon tests.
- Need for reoperation: it will be described with absolute and relative (percentages) frequencies, and it will be compared between groups of interest using Pearson chi-square or Fisher’s exact tests.

#### Subgroup analysis

Subgroup analysis and subgroup effect will be evaluated according to the following variables and categories:

- ○ Age ≥ 75 years (stratification variable) vs age < 75 years
- ○ Preoperative systolic arterial pressure (SAP) at the timing of preoperative visit (stratification variable) ≥ 140 mmHg vs age < 140 years
- ○ RCRI (Revised Cardiac Risk Index - Lee criteria) ≤ 2 *vs* RCRI > 2

### Trial Oversight Committee (TOC) and interim analysis

An independent data and safety Trial Oversight Committee (TOC), consisting of independent ICU trialists/clinicians who have experience in the management of ICU patients and in the conduct, monitoring, and analysis of RCTs, will perform two blinded and planned interim analyses [after enrollment of 106 patients (1th interim analysis) and after the enrollment 420 patients (2nd interim analysis)]. No formal stopping rules will be adopted. A Haybittle–Peto stopping rule (*p* < 0.001) will be used to test for efficacy meaning that if a difference with regard to the primary endpoint with a *p* ≤ 0.001 was detected, the study could be stopped [33], according to the decision of the TOC.

The TOC will be provided with the following masked (as groups 0 and 1) data from the coordinating center:

- ○ Number of patients randomized.
- ○ Number of patients randomized per intervention group.
- ○ Number of protocol violations.
- ○ Number of patients stratified per stratification variable per intervention group.
- ○ Number of events, according to the outcomes, in the two groups.

Based on the evaluation of these outcomes, the T will decide if they want further data from the coordinating center. The TOC can, at any time during the trial, request the distribution of events, including outcome measures and adverse events, according to intervention groups. Further, the TOC can request unblinding of the interventions. Furthermore, the TOC can recommend pausing or stopping the trial if continued conduct of the trial clearly compromises participant safety. The steering committee will make the final decision regarding the continuing, pausing, or stopping of the trial.

## Discussion

The optimal intraoperative MAP strategy for high-risk hypertensive patients scheduled for abdominal elective surgery is unknown, and the HISTAP trial will provide important knowledge on this important topic.

The HISTAP trial is a large trial designed considering the occurrence of postoperative complications obtained by previous trials and has a pragmatic design with all other intraoperative treatments following routine practice to increase external validity. Moreover, the HISTAP trial aims at targeting fluid balance in the context of intraoperative hypotension management by a detailed algorithm of fluid administration, according to hemodynamic parameters.

The trial is monitored according to the standards of GCP [15], and we publish the trial protocol, including details on the outcome assessment and the complete statistical analysis plan. Finally, two interim analyses will be conducted by an independent TOC, which can recommend pausing or stopping the trial if continued conduct of the trial clearly compromises participant safety

The HISTAP trial intervention is not masked for investigators, clinicians, and patients, as blinding of different fluid strategies is not feasible. However, outcome assessors are blinded to allocation. Our trial participants may be subjected to protocol violations, expected to occur more frequently in the treatment group.

## Conclusions

The HISTAP trial is a large pragmatic trial aiming at assessing the optimal intraoperative MAP strategy for high-risk hypertensive patients scheduled for elective abdominal surgery and will provide essential knowledge on this topic.

### Supplementary Information

**Additional file 1.** Supplementary materials.

## Data Availability

Not applicable.
