# Supplementary material for: High versus standard blood pressure target in hypertensive high-risk patients undergoing elective major abdominal surgery: a study protocol for the HISTAP randomized clinical trial
Source: J Anesth Analg Crit Care. 2023 Dec 1;3:50. doi: 10.1186/s44158-023-00133-3 (PMC10691117; doi:10.1186/s44158-023-00133-3)
Supplement: Supplementary file 1 — Additional file 1. Supplementary materials. [file 44158_2023_133_MOESM1_ESM.docx]

**HIgh versus STAndard blood Pressure target in hypertensive high-risk patients undergoing elective major abdominal surgery: the HISTAP randomized clinical trial.**

**Protocol and statistical analysis plan**

**SUPPLEMENTARY MATERIALS**

[General Information 2](#_Toc133568513)

[Trial Registration: 2](#_Toc133568514)

[Study sponsors 2](#_Toc133568515)

[Study coordinator: 2](#_Toc133568516)

[Study Principal Investigator: 2](#_Toc133568517)

[Steering Committee: 2](#_Toc133568518)

[Data monitoring and safety committee (DMSC) 3](#_Toc133568519)

[Study Statistician 3](#_Toc133568520)

[Intraoperative Algorithm of MAP management 4](#_Toc133568521)

[Hypotension treatment protocol – Laparotomic surgery 4](#_Toc133568522)

[Hypotension treatment protocol – Laparoscopic surgery 5](#_Toc133568523)

[Functional hemodynamic test – Mini Fluid Challenge test 6](#_Toc133568524)

[Quality of arterial signal 7](#_Toc133568525)

[Registered variables 8](#_Toc133568526)

[Preoperative records 8](#_Toc133568527)

[Intraoperative records 8](#_Toc133568528)

[Intraoperative hemodynamic parameters 9](#_Toc133568529)

[Daily postoperative records (days 1-7) 9](#_Toc133568530)

[Major complication definition 11](#_Toc133568531)

[Cardiovascular 11](#_Toc133568532)

[Neurological 12](#_Toc133568533)

[Respiratory 13](#_Toc133568534)

[Renal 14](#_Toc133568535)

[Sepsis 14](#_Toc133568536)

[Septic Shock 14](#_Toc133568537)

[Other considerations 16](#_Toc133568538)

[Patient withdrawn 16](#_Toc133568539)

[Dissemination and rules for publication 16](#_Toc133568540)

[Data collection, management and retention 17](#_Toc133568541)

[Status 18](#_Toc133568542)

[Financial compensation for participants 18](#_Toc133568543)

[Bibliography 19](#_Toc133568544)

# General Information

Trial Registration: Clinicaltrials.gov (NCT05637606)

## Study sponsors

- IRCCS Humanitas Research Hospital – IRCCS, Via Alessandro Manzoni, 56 – 20089; Rozzano – Milano (Italy).
- Società Italiana Anestesia, Analgesia, Rianimazione e Terapia Intensiva – SIAARTI - Viale dell'Università, 11 - 00185 - Roma - Partita IVA IT09114630016 - Codice Fiscale 80411520580 - Codice destinatario M5UXCR1 - [siaarti@pec.it](mailto:siaarti@pec.it) – Italy

Study coordinator: Antonio Messina; IRCCS Humanitas Research Hospital – IRCCS, Via Alessandro Manzoni, 56 – 20089; Rozzano – Milano (Italy); Department of Biomedical Sciences, Humanitas University, via Levi Montalcini 4, Pieve Emanuele - Milan, Italy

Study Principal Investigator: Antonio Messina; IRCCS Humanitas Research Hospital – IRCCS, Via Alessandro Manzoni, 56 – 20089; Rozzano – Milano (Italy); Department of Biomedical Sciences, Humanitas University, via Levi Montalcini 4, Pieve Emanuele - Milan, Italy

Steering Committee: Antonio Messina^1,2^; Andrea Cortegiani^3^; Stefano Romagnoli^4^; Giovanni Sotgiu^5^; Federico Piccioni^1,2^; Katia Donadello^6^; Massimo Girardis^7^; Alberto Noto^8^; Salvatore Maurizio Maggiore^9^; Massimo Antonelli^10^; Maurizio Cecconi^1,2^.

^1^Department of Biomedical Sciences, Humanitas University, Pieve Emanuele, Milano, Italy; ^2^IRCCS Humanitas Research Hospital, Rozzano, Milano, Italy; ^3^Department of Surgical Oncological and Oral Science, University of Palermo. Department of Anesthesia Intensive Care and Emergency, Policlinico Paolo Giaccone, Palermo, Italy; ^4^ Department of Anesthesia and Intensive Care, University of Florence, Azienda Ospedaliero-Universitaria Careggi, Florence, Italy; ^5^Clinical Epidemiology and Medical Statistics Unit, Dept of Biomedical Sciences, University of Sassari, Italy; ^6^Unit of Anesthesiology and Intensive Care B, Department of Surgery, Dentistry, Gynecology and Pediatrics, AOUI-University Hospital Integrated Trust of Verona, University of Verona, Verona, Italy; ^7^Department of Anesthesia and Intensive Care, University Hospital of Modena, Modena, Italy; ^8^Department of Human Pathology of the Adult and Evolutive Age "Gaetano Barresi", Division of Anesthesia and Intensive Care, University of Messina, Policlinico "G. Martino", Messina, Italy; ^9^University of Chieti-Pescara and Clinical, Department of Anesthesia, Critical Care and Pain Medicine, SS. Annunziata Hospital, Chieti, Italy; ^10^Institute of Anesthesiology and Resuscitation, Sacred Heart Catholic University, Rome, Italy.

## Data monitoring and safety committee (DMSC)

- Michelle Chew MD, PhD

Department of Anaesthesia and Intensive Care, Biomedical and Clinical Sciences, Linköping University, Sweden; Email: michelle.chew@liu.se

- Daniel De Backer MD, PhD

Department of Intensive Care, CHIREC Hospitals, Université Libre de Bruxelles, Brussels, Belgium; Email: [daniel.de.backer@ulb.be](mailto:daniel.de.backer@ulb.be)

- Chiara Robba MD, PhD

IRCCS Ospedale Policlinico San Martino, Genova, Italy; email: [kiarobba@gmail.com](mailto:kiarobba@gmail.com)

## Study Statistician

Giovanni Sotgiu MD; Clinical Epidemiology and Medical Statistics Unit, Dept of Biomedical Sciences, University of Sassari, Italy; email: gsotgiu@uniss.it

# Intraoperative Algorithm of MAP management

## Hypotension treatment protocol – Laparotomic surgery

*
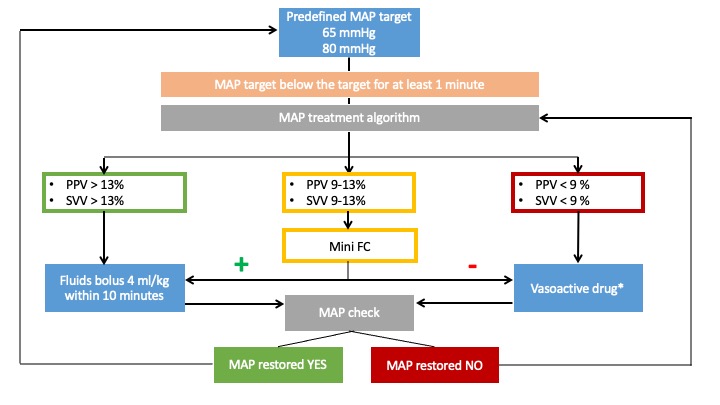
*

PPV, pulse pressure variation; SVV, stroke volume variation; mini-FC, mini fluid challenge; MAP, mean arterial pressure

Rationale: in laparotomic surgery pulse pressure variation (PPV) or stroke volume variation (SVV) maybe used to assess fluid responsiveness and administering a fluid challenge (FC). To avoid the use of vasopressors in hypovolemic patients, the FC is started when PPV or SVV are > 13% (1). On the contrary, PPV or SVV < 9 % indicate fluid non responsiveness, suggesting the use of vasoactive drugs to revert hypotension (as indicated in the protocol – pressure management section). In the “grey zone” of uncertainty of PPV and SVV (between 9% and 13%) a mini FC is given (see further) and a positive min FC response will trigger FC administration, whereas a negative min FC response will trigger vasoactive drug administration. According to previous data from our research group on the efficacy of FC administration in restoring a MAP ≥ 65 mmHg (2), clinicians may consider the use of vasoactive drug during FC administration if MAP is not corrected 3 minutes after FC infusion.

## **Hypotension treatment protocol – Laparoscopic surgery**


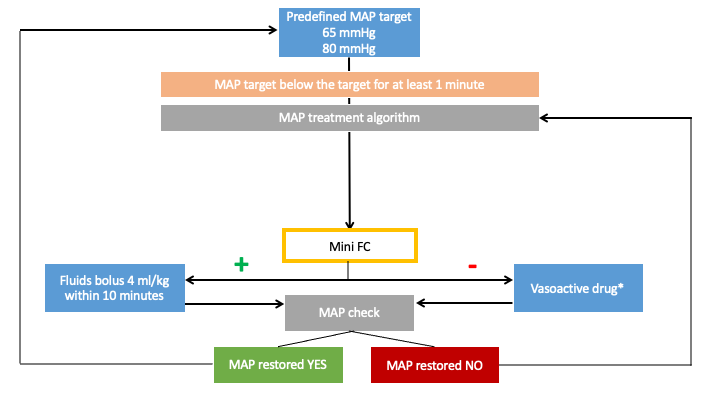


PPV, pulse pressure variation; SVV, stroke volume variation; mini-FC, mini fluid challenge; MAP, mean arterial pressure. Clinician may consider the use of vasoactive drug if MAP is not corrected 3 minutes after FC infusion.

Rationale: the evidence regarding the reliability of PPV or SVV to assess fluid responsiveness during laparoscopy is overall limited (3). In this setting, the mini FC will be used to assess fluid responsiveness.

A positive min FC response will trigger FC administration, whereas a negative min FC response will trigger vasoactive drug administration. According to previous data from our research group on the efficacy of FC administration in restoring a MAP ≥ 65 mmHg (2), clinicians may consider the use of vasoactive drug during FC administration if MAP is not corrected 3 minutes after FC infusion.

This protocol will be also adopted for patients in atrial fibrillation.

## *Functional hemodynamic test – Mini Fluid Challenge test*

The mini FC is a functional hemodynamic test used to predict fluid responsiveness. It is performed by infusion 100 ml of crystalloids in 1 minute (two syringes of 50 ml). The test is considered positive (patient fluid responder) for an increase of the SV or SVI of at least 5%, as compare to baseline values (1). To guide the physician in interpreting this test, we provided a table summarizing the positive results for a range of physiological SV/SVI values (baseline SV or SVI values are expressed as ml or ml/m^2^).

| Baseline  SV /SVI | Positive  mini-FC | Baseline  SV /SVI | Positive  mini-FC | Baseline  SV /SVI | Positive  mini-FC | Baseline  SV /SVI | Positive  mini-FC |
| --- | --- | --- | --- | --- | --- | --- | --- |
| 15 | 16 | 31 | 33 | 47 | 49 | 63 | 66 |
| 16 | 17 | 32 | 34 | 48 | 50 | 64 | 67 |
| 17 | 18 | 33 | 35 | 49 | 51 | 65 | 68 |
| 18 | 19 | 34 | 36 | 50 | 53 | 66 | 69 |
| 19 | 20 | 35 | 37 | 51 | 54 | 67 | 70 |
| 20 | 21 | 36 | 38 | 52 | 55 | 68 | 71 |
| 21 | 22 | 37 | 39 | 53 | 56 | 69 | 72 |
| 22 | 23 | 38 | 40 | 54 | 57 | 70 | 74 |
| 23 | 24 | 39 | 41 | 55 | 58 | 71 | 75 |
| 24 | 25 | 40 | 42 | 56 | 59 | 72 | 76 |
| 25 | 26 | 41 | 43 | 57 | 60 | 73 | 77 |
| 26 | 27 | 42 | 44 | 58 | 61 | 74 | 78 |
| 27 | 28 | 43 | 45 | 59 | 62 | 75 | 79 |
| 28 | 29 | 44 | 46 | 60 | 63 | 76 | 80 |
| 29 | 30 | 45 | 47 | 61 | 64 | 77 | 81 |
| 30 | 32 | 46 | 48 | 62 | 65 | 78 | 82 |

## *Quality of arterial signal*

We aimed at ensuring the quality of the arterial signal and, hence, of the continuous cardiac output monitoring by suggesting a systematic 5-steps approach to the atrial catheter, as recently proposed by Saugel et al. (4)

- Step 1: Catheter insertion sites
- Step 2: Choosing the type of arterial catheter
- Step 3: Placement of the arterial catheter
- Step 4: Leveling and zeroing of the pressure transducer
- Step 5: Checking the quality of the arterial blood pressure waveform—morphology and artifacts

A copy of this comprehensive review (4) has been given to all the participating centers and used as guideline for positioning and monitoring arterial signal.

# Registered variables

## Preoperative records

- Sex
- Age
- Weight
- Height
- Years from hypertension starting treatment (as declared by the patient)
- SAP at the time of preoperative visit (mean of three consecutive measurements).
- Scores at baseline: Charlson Index; (Global Initiative for Chronic Obstructive Lung Disease) GOLD Scale; Clinical Frailty Scale; ASA Score; New York Heart Association (NYHA) Objective Assessment and classification; Metabolic equivalents (MET); RCRI (Revised Cardiac Risk Index - Lee criteria); NSQIP score.
- Preoperative Labs (blood count, biochemistry and coagulation) – according to the local preoperative screening for high-risk surgical patients.

## Intraoperative records

- Date (XX/YY/20ZZ) and hour (XX:YY)
- Procedure description and type
- Duration of the operation SKIN-SKIN (min)
- Duration of the operation INDUCTION / AWAKENING (min)
- Timing from induction to hemodynamic monitoring (and type of hemodynamic monitoring used)
- Protocol violation and reason for violation
- Type of discharge from the surgical block
- Associated regional anesthesia – type and dose
- Intraoperative drugs (induction) – type and dose
- Intraoperative drugs (maintenance) – type and dose
- Type (crystalloids or colloids) and volume (ml) of intraoperative fluids
- Total blood loss (ml) and blood transfusion (Y/N, number of packages).
- Total diuresis (ml)
- End-of-procedure fluid balance (ml)
- Vasopressor use (dose and starting time)
- Number of hypotensive episodes
- Lowest MAP recorded
- Ventilator setting (Tidal Volume, PEEP, FiO_2_ , respiratory rate)

## Intraoperative hemodynamic parameters

- SAP / MAP / diastolic arterial pressure (DAP) / heart rate (HR) / cardiac output (CO) / strike volume (SV) at minutes 3 - 6 and 9 after induction
- SAP / MAP / DAP / HR / CO / SV every 10 minutes during each hour of operation

## Daily postoperative records (days 1-7)

- Patient condition:
- Alive and hospitalized
- Died
- Discharged at home
- Transferred to another hospital
- Cardiovascular major events (see further)
- Respiratory major events (see further)
- Infective major events (see further)
- Neurological major events (see further)
- acute kidney injury (AKIN) stage at day 1-2-3 and 7 (if patient not discharged)
- Variables for Sequential Organ Failure Assessment (SOFA) score at day 1-2-3 and 7 (if patient not discharged)
- Lowest MAP (as reported in the ward vital parameters record)
  Lowest oxygen saturation (SaO2) (as reported in the ward vital parameters record)
  Highest HR (as reported in the ward vital parameters record)
  Lowest HR (as reported in the ward vital parameters record)
  Lowest Temperature (as reported in the ward vital parameters record)
  Highest Temperature (as reported in the ward vital parameters record)
- Urine output (as reported in the ward vital parameters record)
- Postoperative Labs (blood count, biochemistry, coagulation and troponin) – standard laboratory values.

# Major complication definition

## Cardiovascular

- Acute heart failure (5) defined as a syndrome consisting of cardinal symptoms (e.g. breathlessness, ankle swelling, and fatigue) that may be accompanied by signs (e.g. elevated jugular venous pressure, pulmonary crackles, and peripheral oedema), due to a structural and/or functional abnormality of the heart that results in elevated intracardiac pressures and/or inadequate cardiac output.
- Myocardial injury after non-cardiac surgery (MINS). MINS was defined as any myocardial infarction (as defined below), and any elevated troponin (i.e., a value higher than the local laboratory threshold) judged to be due to myocardial ischemia (i.e., without evidence of a nonischemic etiology [e.g., chronic elevation, pulmonary embolism, sepsis, cardioversion]) (6).
  - Non-high sensitivity fourth-generation troponin T ≥ 30 ng/L
  - High sensitivity troponin T from 20 to < 65 ng/L with an absolute change of ≥ 5 ng/L
  - High sensitivity troponin T ≥ 65 ng/L
- Myocardial infarction (7) diagnosis, requiring any one of the following criteria .
  - Detection of a rise or fall of a cardiac biomarker (preferably troponin) with at least one value above the 99th percentile of the upper reference limit (URL) together with evidence of myocardial ischemia with at least one of the following:
    - Ischemic signs or symptoms (i.e., chest, arm, neck, or jaw discomfort, shortness of breath, or pulmonary edema)
    - New onset of atrial fibrillation or persistent arrythmia requiring pharmacological treatment (confirmed by ECG and/or by specialist).
    - Development of pathologic Q waves present in any two contiguous leads that are ≥ 30 milliseconds.
    - New or presumed electrocardiography (ECG) changes indicative of ischemia (i.e., ST segment elevation [≥2 mm in leads V1-3 OR ≥ 1 mm in the other leads], ST segment depression [≥1 mm], or symmetric inversion of T waves ≥1 mm) in at least two contiguous leads.
    - New left bundle branch block (LBBB).
    - New cardiac wall motion abnormality on echocardiography or new fixed defect on radionuclide imaging.
    - Identification of intracoronary thrombus on angiography or autopsy.
- Bradycardia (persistent heart rate <40 beats/min with symptoms and needing pharmacological or non-pharmacological treatment)
- Symptomatic proximal deep venous thrombosis (DVT) required:
  - symptoms or signs that suggested DVT (e.g., leg pain or swelling)
  - thrombosis involving the popliteal vein or more proximal veins for leg DVT or axillary or more proximal veins for arm DVTs.
  - Any of the following defined evidence of vein thrombosis:
    - A persistent intraluminal filling defect on contrast venography (including on CT).
    - Non compressibility of one or more venous segments on B mode compression ultrasonography.
    - A clearly defined intraluminal filling defect on doppler imaging in a vein that cannot have compressibility assessed (e.g., iliac, inferior vena cava, subclavian).
- Peripheral arterial and venous thrombosis (any district):
  - clear evidence of abrupt occlusion of a peripheral artery or vein documented by any documented report (echo, intraoperative surgical finding, CT scan, autopsy) and inducing tissue ischemia (bowel, muscle, skin etc).

## Neurological

- Stroke (8) confirmed by specialist neurologist and/or from CT scan/MRI documentation.
  - Ischemic: An episode of neurological dysfunction caused by focal cerebral, spinal, or retinal infarction.
  - Hemorragic: A focal collection of blood within the brain parenchyma or ventricular system that is not caused by trauma.
  - Not otherwise specified: An episode of acute neurological dysfunction presumed to be caused by ischemia or hemorrhage, persisting ≥24 hours or until death, but without sufficient evidence to be classified as one of the above.
- Subarachnoid hemorrhage: Bleeding into the subarachnoid space (evidence from CT scan / MRI).
- Cerebral venous thrombosis: Infarction or hemorrhage in the brain, spinal cord, or retina because of thrombosis of a cerebral venous structure (evidence from CT scan / MRI).
- Seizure: abrupt onset of focal or generalized experiential, motor, sensory or cognitive phenomena, in absence of another etiology for the event, confirmed by specialist neurologist.
- Acute delirium requiring pharmacological treatment.
  - The presence of delirium will be determined by means of structured interviews preoperatively and on the first postoperative days 1-3 and 7, using 4 A's test (4AT) (9). To ensure consistency in the evaluation, each patient will be evaluated by the same research assistant (blinded to the study allocation) for all three interviews. All cases of incident delirium were validated by a second senior investigator.

## Respiratory

- Acute respiratory distress: Clinical signs suggestive of intense respiratory muscle work and/or labored breathing, such as use of accessory respiratory muscles, paradoxical motion of the abdomen, or intercostal retraction
- Hypoxemia with or without acute respiratory distress (PaO2 <60 mmHg or SpO2 <90% on room air for more than 30 minutes).
- Need for invasive, non-invasive ventilation or HFNC for acute respiratory distress.
- Acute respiratory distress syndrome (10).
- Pulmonary edema: documented evidence of respiratory distress or impaired oxygenation and radiological evidence of pulmonary edema.
- Pulmonary embolism:
  - The diagnosis of symptomatic pulmonary embolism required symptoms (e.g., dyspnea, pleuritic chest pain) or signs (e.g., hypoxia, increased work of breathing) and any one of the following:
    - An intraluminal filling defect of segmental or larger artery on a helical CT scan.
    - Echographic pattern of right ventricle acute dysfunction assessed by an expert of echocardiography.
    - A positive diagnostic test for deep venous thrombosis (e.g., positive compression ultrasound) and one of the following:
      - A. non-diagnostic (i.e., low or intermediate probability) ventilation/perfusion lung scan; or
      - B. non-diagnostic (i.e., subsegmental defects or technically inadequate study) helical CT scan.

## Renal

- AKI and AKI stages are defined as follows, according to the AKIN classification/staging system of acute kidney injury (11):


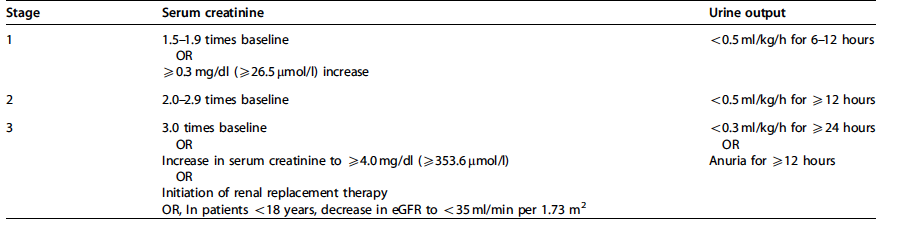


## Sepsis

The Third International Consensus Definitions Task Force (12) defined sepsis as a “life-threatening organ dysfunction due to a dysregulated host response to infection.” Based on the Third International Consensus Definitions for Sepsis and Septic Shock (Sepsis-3) criteria, sepsis required a quick Sequential Organ Failure Assessment (qSOFA) Score ≥ 2 points due to infection. The qSOFA included the following items and scoring system:

- Glasgow Coma Scale (GCS) score of 13 or less (1 point)
- Systolic blood pressure of 100 mm Hg or less (1 point)
- Respiratory rate of 22 breaths/min or more (1 point).

## Septic Shock

- Septic shock definition according to the SSC guidelines 2021 (13)

Source of sepsis/septic shock: certain/uncertain

- - - Anastomotic leak and/or perianastomotic abscess (confirmed by CT scan and from specialist) requiring or not reoperation
    - Pneumonia / HAP - VAP (hospital/ventilatory associated pneumonia see below). Criteria for postoperative pneumonia: the presence of new and/or progressive pulmonary infiltrates on chest radiograph / CT scan plus two or more of the following criteria:

a) Fever ≥38.5° or hypothermia <36°

b) Leukocytosis ≥12000 WBC/mm^3^ or leukopenia <4000 WBC/mm^3^

- - VAP definition: A pneumonia where the patient is on mechanical ventilation for >2 calendar days on the date of event, with day of ventilator placement being Day 1.
  - HAP is defined as pneumonia that occurs 48 hours or more after admission, which was not incubating at the time of admission.
- Blood stream infection
  - - - Central Venous Catheter related
      - Midline/PICC related
    - Urinary infection
    - Abdominal infection
    - CNS infection
    - Other infection (specify)

# Other considerations

## Patient withdrawn

Patients may be withdrawn from the study if the patient withdraw consent. Patients who will withdraw from the study will be followed up until hospital discharge, according to routine clinical practice in each participating center. Patients will be asked for permission to use data obtained prior to withdrawal and to obtain data for the primary outcome measure. If this is achieved the patient will be included in the final analyses. If the patient declines, all data from that patient will be destroyed and a new patient will be randomized to obtain the full sample size.

## Dissemination and rules for publication

The HISTAP trial is registered at [www.clinicaltrials.gov](http://www.clinicaltrials.gov) (NCT05637606). After the completion of the HISTAP trial, all trial results will be submitted to a peer‐reviewed medical journal irrespective of the direction of the results. We will adhere to the CONSORT statement including the accountability of all patients screened. The Steering Committee will grant authorship depending on personal involvement according to the Vancouver definitions. The listing of authors will be as follows: A Messina (principal investigator) will be responsible for the writing of the manuscript and will be the first author, and the next authors will be the other members of the Steering Committee according to the number of included patients per study site, then trial site investigators dependent on the number of included patients per site, M Cecconi will appear as the last author and then ‘for the SIAARTI HISTAP study group’. The Principal Investigator is responsible for the conduct of the study and data processing. In agreement with ICH-GCP, the Principal Investigator agrees to produce a yearly report on the study and publish all data generated from this study irrespective of results. The Principal Investigator will ensure that data are properly reported, and research findings are disseminated responsibly. Data dissemination and communication through scientific publications and/or presentations at congresses and conferences, as well as participation in multicentre studies, will follow statistical analysis of anonymized data. Publication of the data takes place in an aggregated form only. Information about individual patients will not be published or shared. A group authorship (“SIAARTI Study Group”) will be created, including all the investigators of the participating centres. The number of investigators for each center will be related to the number of the included patients and to the quality of data collection. Every 10 patients with complete records, two investigators will be assured to the respective center. Every 25 patients with complete records, three investigators will be assured to the respective center. Every 50 patients with complete records, six investigators will be assured to the respective center.

Side studies will be allowed if supported by the Steering committee.

## Data collection, management and retention

Data collection serves a scientific purpose. The data will be generated in the participating centres and recorded via a web application on the European servers of the Italian Society of Anaesthesiology, Analgesia, Resuscitation and Intensive Care (SIAARTI) using the study management software RedCap (Research Electronic Data Capture) (14, 15).  REDCap is a secure, web-based software platform designed to support data capture for research studies, providing 1) an intuitive interface for validated data capture; 2) audit trails for tracking data manipulation and export procedures; 3) automated export procedures for seamless data downloads to common statistical packages; 4) procedures for data integration and interoperability with external sources. Data will be recorded in the e-CRF only using coding procedures described in a separate document to comply with data protection applicable laws, including EU GDPR. A coding key reporting patient’s name, surname, and date of birth will be filed only in local site files together with a subject screening and enrolment log.  In cases where a medical institution is interested in participation, for each authorized user an account is created in the RedCap platform.

Data will be uploaded using an encrypted data connection (HTTPS) throw a web browser or mobile app and stored in an encrypted database. To ensure pseudonymized data analysis, each patient will be assigned a unique Subject ID (Patient Identification Number). RedCap is a secure web application for building and managing online databases. By using a hierarchical, role-based access concept, unauthorized access to the data will be prevented. Access to RedCap will be granted only to data collecting staff of participating centres, in accordance with the procedures outlined in the present protocol. These persons are bound to secrecy. Principal Investigator and monitoring team will maintain adequate and accurate the clinical report forms. SIAARTI and the steering committee, on behalf of the investigators, have the right to use all data that are pooled in the databank for scientific purposes. Investigators will be regularly informed about ongoing study activities. The steering committee and the SIAARTI Study Group have the right to access the data, pooled in the databank, for research purposes after the research project has been terminated, and with the approval of the SIAARTI Scientific Committee.

A copy of the electronic databank will be kept in SIAARTI databases and preserved for 5 years for subsequent use by SIAARTI and Steering Committee.  Data will remain property of Principal Investigator and Humanitas Research Hospital and of SIAARTI.

## Status

The Trial was initiated on March 6th 2023 and the enrollment is expected to be completed within 2 years.

## Financial compensation for participants

There will be no financial compensation for participants.

# Bibliography

1. Messina A, Dell'Anna A, Baggiani M, Torrini F, Maresca GM, Bennett V, et al. Functional hemodynamic tests: a systematic review and a metanalysis on the reliability of the end-expiratory occlusion test and of the mini-fluid challenge in predicting fluid responsiveness. Critical care. 2019;23(1):264.

2. Messina A, Colombo D, Lionetti G, Calabro L, Negri K, Robba C, et al. Pressure response to fluid challenge administration in hypotensive surgical patients: a post-hoc pharmacodynamic analysis of five datasets. Journal of clinical monitoring and computing. 2022.

3. Chen J, Zhao S, Zhu Q. Reliability of stroke volume or pulse pressure variation as dynamic predictors of fluid responsiveness in laparoscopic surgery: a systematic review. Journal of clinical monitoring and computing. 2022.

4. Saugel B, Kouz K, Meidert AS, Schulte-Uentrop L, Romagnoli S. How to measure blood pressure using an arterial catheter: a systematic 5-step approach. Critical care. 2020;24(1):172.

5. McDonagh TA, Metra M, Adamo M, Gardner RS, Baumbach A, Bohm M, et al. 2021 ESC Guidelines for the diagnosis and treatment of acute and chronic heart failure. European heart journal. 2021;42(36):3599-726.

6. Ruetzler K, Smilowitz NR, Berger JS, Devereaux PJ, Maron BA, Newby LK, et al. Diagnosis and Management of Patients With Myocardial Injury After Noncardiac Surgery: A Scientific Statement From the American Heart Association. Circulation. 2021;144(19):e287-e305.

7. Thygesen K, Alpert JS, Jaffe AS, Chaitman BR, Bax JJ, Morrow DA, et al. Fourth universal definition of myocardial infarction (2018). Eur Heart J. 2019;40(3):237-69.

8. Powers WJ, Rabinstein AA, Ackerson T, Adeoye OM, Bambakidis NC, Becker K, et al. Guidelines for the Early Management of Patients With Acute Ischemic Stroke: 2019 Update to the 2018 Guidelines for the Early Management of Acute Ischemic Stroke: A Guideline for Healthcare Professionals From the American Heart Association/American Stroke Association. Stroke. 2019;50(12):e344-e418.

9. Saller T, MacLullich AMJ, Schafer ST, Crispin A, Neitzert R, Schule C, et al. Screening for delirium after surgery: validation of the 4 A's test (4AT) in the post-anaesthesia care unit. Anaesthesia. 2019;74(10):1260-6.

10. Ferguson ND, Fan E, Camporota L, Antonelli M, Anzueto A, Beale R, et al. The Berlin definition of ARDS: an expanded rationale, justification, and supplementary material. Intensive care medicine. 2012;38(10):1573-82.

11. Mehta RL, Kellum JA, Shah SV, Molitoris BA, Ronco C, Warnock DG, et al. Acute Kidney Injury Network: report of an initiative to improve outcomes in acute kidney injury. Critical care. 2007;11(2):R31.

12. Singer M, Deutschman CS, Seymour CW, Shankar-Hari M, Annane D, Bauer M, et al. The Third International Consensus Definitions for Sepsis and Septic Shock (Sepsis-3). JAMA. 2016;315(8):801-10.

13. Evans L, Rhodes A, Alhazzani W, Antonelli M, Coopersmith CM, French C, et al. Surviving sepsis campaign: international guidelines for management of sepsis and septic shock 2021. Intensive care medicine. 2021;47(11):1181-247.

14. Harris PA, Taylor R, Thielke R, Payne J, Gonzalez N, Conde JG. Research electronic data capture (REDCap)--a metadata-driven methodology and workflow process for providing translational research informatics support. J Biomed Inform. 2009;42(2):377-81.

15. Harris PA, Taylor R, Minor BL, Elliott V, Fernandez M, O'Neal L, et al. The REDCap consortium: Building an international community of software platform partners. J Biomed Inform. 2019;95:103208.
